# Characteristics, opportunities, and challenges of osteopathy (COCO) in the perceptions of osteopaths in Germany, Austria, and Switzerland: a metasynthesis

**DOI:** 10.1186/s40001-024-02199-3

**Published:** 2024-12-18

**Authors:** Jonas Manschel, Jan Porthun, Jean Marie A. T. Beuckels, David Martin

**Affiliations:** 1 Institute of Integrative Medicine, Department of Medicine, Health Faculty, University Witten/Herdecke, Herdecke, Germany; 2 Norwegian University of Science and Technology, Campus Gjøvik, Gjøvik, Norway; 3 Department of Osteopathy, Faculty of Health and Social Sciences, Hochschule Fresenius, Munich, Germany; 4 Tübingen University Children’s Hospital, Tübingen, Germany

**Keywords:** Osteopath, Osteopathic practitioner, Osteopathy, Osteopathic profession, Health care system, Germany, Austria, Switzerland

## Abstract

**Background:**

The practice of osteopathy in Europe is not uniformly regulated. Even despite the topographical and cultural proximity, the regulation of the osteopathic profession also differs in the German-speaking countries. In contrast to Germany and Austria, both without any recognized osteopathic profession, Switzerland has already created legal regulations for the practice of osteopathy. The aim of this analysis and the project itself is to gain knowledge about the characteristics, challenges and opportunities of osteopaths in German-speaking countries.

**Methods:**

The COCO project examines osteopaths in Germany, Austria, and Switzerland, considering their view on the practice of osteopathy. Within the framework of a metasynthesis, a systematic literature search was conducted first to identify further relevant studies. Subsequently, a qualitative synthesis was followed after having applied the inclusion and exclusion criteria to the literature found.

**Results:**

This study was able to draw on a total of 30 content-analyzed interviews with osteopaths. It was possible to gain deeper insights into the characteristics, challenges and opportunities of osteopaths in German-speaking countries.

Challenges were diagnosed, for example, regarding professional identity, as well as in the intraprofessional conflicts and the question of standards in training and research. The chances were described as leading a fulfilling professional life and meeting a great interest in osteopathy among the population.

**Conclusions:**

Regarding the characteristics of osteopathy in Germany, Austria and Switzerland, it can be said that osteopaths have difficulties to define osteopathy. The resources mentioned most often are manual work on the patient, a holistic treatment approach and generous treatment durations. Lecturers and prominent figures can be seen as role models and greatly influence the perspective of osteopaths. The central challenge is the problem of identity among osteopaths. In addition, different training programs and qualifications exist, which also presents the greatest country-specific differences. The satisfaction of osteopaths in their work and the extreme demand for osteopathy in society should be seen as an opportunity for the field.

**Supplementary Information:**

The online version contains supplementary material available at 10.1186/s40001-024-02199-3.

## Background

According to the WHO, osteopathy offers various therapeutic approaches for the treatment of diseases, health maintenance and stimulation of self-healing processes. It is firmly anchored in its own principles and corresponding philosophy [1]. A study of the international profile of osteopathic care in ten countries shows that osteopaths are highly trained, independent medical professionals who mainly treat people with musculoskeletal complaints, usually of the spine, that have existed for more than a month [2]. In practice, however, major differences can be detected between individual osteopathic practitioners and their treatment paradigms [3]. In addition, legal regulation in the 27 European member states is inconsistent. Currently in Europe, twelve countries have regulated the professional practice of osteopathy [4]. Not least because of the different jurisdictions, it is impossible to speak of a common European osteopathic identity [5]. Having a closer look at the professional regulations of osteopathy practice in German-speaking countries reveals clear differences despite their topographical proximity and cultural similarities.

In Germany, osteopathic practitioners with prior training in medicine, physiotherapy or as a naturopath (or “alternative practitioners”, dt. Heilpraktiker) practice osteopathy [6]. Currently, no occupational laws for osteopaths exists. However, case law has severely restricted osteopaths with prior training in physiotherapy since the 2010s [7, 8]. The first study of a profile of German osteopathic practitioners dates from 2019. The data from Dornieden show that osteopaths and osteopathic physicians in Germany use a variety of osteopathic treatment methods and that patients report with a variety of complaints, illnesses and symptoms. The heterogeneous nature of the osteopathic community with different professional backgrounds and their impact on professional identity is described [9].

In Austria, osteopathic practitioners with prior training in medicine or physiotherapy work without a specific occupational law for osteopathy, but only physicians can practice osteopathy without restrictions. According to their legal basis (Medizinisch-technische Dienste-Gesetz), non-physician professional groups depend on a physician's referral [10]. In 2022, a quantitative questionnaire study on the characteristics of Austrian osteopathic practitioners was conducted. The "average Austrian osteopath" was described as female, aged between 40 and 49, self-employed, as having completed a part-time training with a master's degree and previous physiotherapy training [11].

Switzerland is the only one of the German-speaking countries to have recognized osteopathy as profession as it has a professional law, including the implementation of the profession in the healthcare system. Moreover, the *Gesundheitsberufegesetz* (GesBG) and the *Ausführungsrecht* have been in force since the beginning of 2020. These laws provide regulated standards for the training for a total of seven healthcare professions [12]. However, after a 5-year transitional period, some practitioners, whose qualifications are not in line with these new laws, are now facing a professional ban [13].

In view of these highly different legal circumstances, the *Characteristics, Opportunities, and Challenges of Osteopathy* study project (COCO) aims to investigate the characteristics, possibilities and opportunities of osteopathy in Germany, Austria and Switzerland. So far, quantitative studies have focused exhaustively on the population of osteopaths with regard to work status, training, professional identity or characteristics of clinical practice such as the average patient profile and the use of diagnostic and treatment modalities [11, 14]. The objective was to conduct studies with qualitative designs to gain a more detailed understanding of the profession of osteopathy in German-speaking countries. The following explanations intend to review qualitative studies to allow for new theoretical insights from the accumulation of study results.

This study forms a part of the COCO study project. The COCO study project is transnational and examines osteopaths in the German-speaking countries and their view on the practice of osteopathy. The detailed mixed-methods design consists of both qualitative and quantitative partial studies. The exact project procedure was developed and published in 2019 in the form of a study protocol [15]. It is an exploratory design involving qualitative study designs with small number of respondents to form hypotheses first and then checking their general validity for the entirety of osteopathic practitioners by means of a quantitative survey in a second step [16]. Three of these partial studies were conducted as final theses at the *Osteopathie Schule Deutschland* in cooperation with *Dresden International University* [17–19]. Two of these studies were conducted with osteopaths from Germany. One study surveyed osteopathic practitioners in Switzerland and has already been published in parts in 2020 [20]. A further study with subjects from Austria was completed and has been published in 2024 [21]. The availability of studies from all three German-speaking countries finally allows to compare the results, taking into account further literature, to gain further insights through synthesis.

## Methods

### Qualitative metasynthesis

The research on qualitative studies dealing with characteristics of osteopathic profession in Germany, Austria and Switzerland provides the base for this work. A metasynthesis is advisable if only few studies on a research topic are available or if, as expected in this case, the number of studies found through literature research is highly limited. Results are then summarized and interpreted and can thus contribute to a deeper understanding of the research topic [22].

### Systematic literature research

The aim of the literature search was to identify further reading in addition to the four studies already conducted as part of the COCO study project. To allow for a systematic search, the following inclusion and exclusion criteria were formulated in advance (see Table 1). We excluded quantitative studies or systematic reviews in our synthesis, because they did not align with our identified purpose.

**Table 1:** Inclusion and exclusion criteria

Inclusion criteria
Studies, theses and dissertations that are either found using a keyword-based search in selected medical databases or/and that were conducted within the COCO study project
Studies, theses and dissertations written in English or German
Studies, theses and dissertations that must be accessible in full text
Exclusion criteria
Studies, theses and dissertations that either have a different study design or/and do not match the research questions thematically
Studies, theses and dissertations that were published over 10 years ago

A concept matrix was developed on the basis of the research questions of the COCO study project (see Table 2). A detailed list of the search strings used for the respective platforms can be found in the appendix (see Supplementary Materials “Bibliographic Databases Search”).

**Table 2:** Concept matrix COCO-study-metasynthesis

	Topic 1	Topic 2	Topic 3
Keywords (German)	Charakteristika	Möglichkeiten	Herausforderungen
Keywords	Characteristics	Opportunities	Challenges
Related terms (German)	Definition, Identität, Überzeugungen	Ausübung	Schwierigkeiten
Related terms	Definition, identity, attitude, beliefs	Practice	Difficulties
Broader term (German)	Beruf, Berufsbild, Ausbildung		
Broader term	Profession, profile, training		

**Fig. 1:**
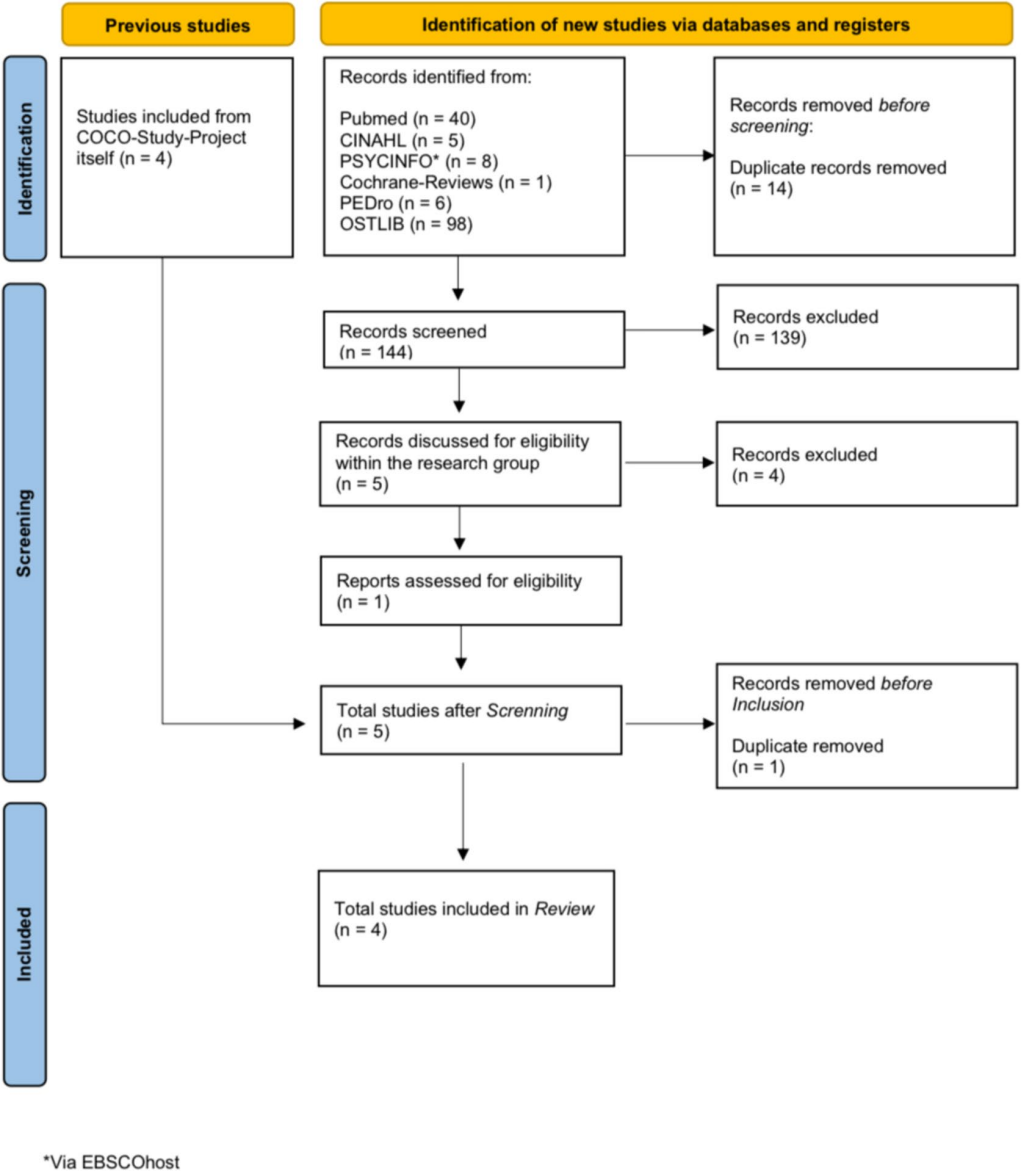
Prisma 2020 flow diagram

The databases Pubmed, CINAHL, Psycinfo, Cochrane Reviews, PEDro and OSTLIB, which are considered as relevant in the medical-therapeutic field, were searched (JM). The literature search was then repeated independently by another person using defined search parameters (JP). The identified literature was summarized in a table (see appendix), and a decision on inclusion or exclusion was made after a closer look at the content. The literature search was conducted independently by two authors (JM and JP). The individual evaluations were then compared to each other, differences between the two evaluators were discussed (JM and JP) and a consensus on the literature regarded as useful for the research project was reached. The complete list of reviewed literature is provided by the authors in the appendix (see supplementary materials—“Reviewed Literature”). After applying the inclusion and exclusion criteria and removing the duplicates, the literature search only identified one study from the COCO project itself that fitted the profile (see Fig. 1) [23].

## Results

All four studies included in the synthesis contain interviews with osteopaths from German-speaking countries and a subsequent content analysis. Two studies focus on osteopaths in Germany and two studies focus on osteopaths from each Austria and Switzerland (see Fig. 1). As a result, this metasynthesis can therefore draw on the content analysis results of interviews with a total of 30 osteopaths (see Table 3).

**Table 3:** Participants' charateristics included studies

	Manschel et al. [21]	Süß [19]	Bücker [18]	Meier and Porthun [20]
Study design	Guideline-based interviews with osteopaths	Guideline-based interviews with osteopaths	Guideline-based interviews with osteopaths	Guideline-based interviews with osteopaths
Data analysis	Content analysis according to Mayring	Content analysis according to Mayring	Content analysis according to Mayring	Content analysis according to Mayring
Country	Austria	Germany	Germany	Switzerland
Number of participants (*n*)	10	4	9	7
Gender (*n*)	Male 4 Female 6	No information On gender distribution	Male 5 Female 4	Male 4 Female 3
Prior training (*n*)	Physician 3 Physical therapist 7	Physical therapist 1 Naturopaths 2 Other 1	Physician 1 Physical therapist 6 Naturopaths/other 2	Physical therapist 4 Other/non 3
Interviewer's expertise	M.Sc. Osteopathy	Master's student in osteopathy	Master's student in osteopathy	Master's student in osteopathy

A closer look at the four studies in question reveals a similar way of presenting results. All of the studies initially structure their results deductively according to the research questions of the COCO project. The authors of the partial studies assigned relevant text passages from the respective interviews to the research questions of characteristics, challenges, and opportunities of osteopathy. In addition, further sub-codes were created deductively and inductively to categorize and evaluate statements uttered by the respondents. The entire sub-codes of the four studies were listed, compared, summarized and differentiated for this metasynthesis, as shown exemplarily below (see supplementary materials “List of selected codes”).

### Characteristics of osteopathy

#### Definition of osteopathy

In all studies, many respondents were not able provide a consistent definition of osteopathy. Many respondents even expressed difficulties in precisely describing their profession.

*"Yes, it's really a difficult question." (O_1, item 35)* [21]

*"For me, osteopathy [is], phuu, difficult. It's so huge and*

*Multi-faceted." (O7, item 26)* [17]

*"I'll have to think about that (laughs)." (Interview 06, item 35)* [18]

Nevertheless, recurring patterns in the explanations given by the respondents from German-speaking countries can be identified. For example, concrete actions (descriptions of treatments) and first-contact practice were often used to explain what differentiates osteopathy from allied health profession (e.g., physiotherapy).

The respondents also attempted to provide a definition of osteopathy by drawing a distinction between osteopathy and other professions and using third-party definitions:

*"As far as I know, [Andrew Taylor] Still never provided a concrete definition because I think he felt that a definition was far too narrow for osteopathy. I think osteopathy is much bigger than could be described in a precise, concise definition." (Interview 02, item 48)* [18]

Furthermore, the philosophy of osteopathy, various osteopathic concepts or models of thoughts, the holistic nature of the treatment method and the activation of the patient's self-healing powers are referred to multiple times.

Only one osteopath from Switzerland refers to the official definition of the professional organizations and the WHO, respectively (O7, item 26) [17].

One possible reason for the heterogeneous explanations may lie in the different educational backgrounds (O2, item 18). Historical, political and cultural developments in the different countries should also be considered (O1, item 23) [19].

#### Qualities of osteopathy

For the description of the characteristics of osteopathy, many interviewees start with the most obvious: osteopathic treatment is carried out with the hands. Due to years of practicing osteopathy on patients, they characterize osteopaths by their good palpation skills [17, 18].

*„[T]ouch is something that is set in our mind from the start brought with effective touch, with healing. Probably our first memories of healing is our parents soothing or rubbing against a pain, or giving a kiss, so I think all this touch relationship to our health condition is anchored into our system. So naturally, there’s an inclination of saying, if I feel pain somewhere, I’m expecting someone to touch it “ (O5, item 61)* [17]

The holistic nature of the treatment method is often emphasized as being a unique feature of osteopathy. For some participants, this includes both the therapy of all three systems (parietal, visceral, cranial) and the concentrated search for the cause of a symptom in contrast to other local manual treatment approaches [17–19]. The activation of the patient's self-healing powers as an overriding goal of osteopathic intervention is also mentioned multiple times [17, 18].

The generous consultation time is also repeatedly described as a strength of osteopathy. The osteopaths surveyed often invest more time in their patients than members of other medical professions. This greater amount of information during consultation time may significantly contribute to the success of osteopathic treatment [17]. One respondent did not want to exclude a placebo effect in this context (O1, item 105) [19]. In addition to the generous consultation time, the individual approach to the patient presents a central quality of osteopathy.

*"I think it's important to take one’s time for the patient. Many methods are becoming shorter and shorter in terms of consultation times and you no longer feel that you are being taken seriously. I think it's a strength of osteopathy that you really take this time and listen to the patient and give them the opportunity to explain themselves." (O1, item 100)* [17]

The mature patient is also granted a say in the therapy within osteopathic practice. For example, some interviewees attest a great importance to empathetic skills in patient–therapist interaction [17, 18].

#### Differentiation from other professions in the healthcare sector

The respondents particularly often differentiate osteopathic work from physiotherapy, while many of the osteopaths underwent physiotherapy training themselves. This presents another finding revealed by the COCO studies.

*“For me it is, a manual therapy in which one not only treats joints, bones and muscles, as perhaps in physiotherapy, but in which one also treats internal organs, in which one treats the vascular system (…).” (O1, item 24)* [17]

In detail, a distinction between legal practice and the duty to follow instructions by physicians is made consciously. Here, physiotherapists must generally follow the physicians' prescriptions with regard to treatment methods and treatment frequency. The difficulties of billing or reimbursement of treatment costs by health insurance companies are also mentioned [17, 18].

Another difference to other medical professions mentioned by the participants refers to their palpatory skills, which enable precise tactile findings. They regard this as a contrast to the device-based diagnostics mainly used in conventional medicine (O2, item 41) [17]. However, the respective professional training/education must also be taken into account here. If an osteopath is not a trained physician at the same time, s/he is not authorized to order or even perform radiological examinations, so s/he has to rely on their palpation findings. Thus, the trained hands of an osteopath and its palpation findings could be a unique feature of the profession [17–19].

Treatment of chest or abdominal organs as well as the skull was mentioned by the interviewed osteopath as another unique feature of osteopathy. In contrast, treatment in physiotherapy or chiropractic is often limited to the musculoskeletal (parietal) system[17, 18].

*"A physiotherapist focuses on the musculoskeletal system." (O3, item 34)* [17]

Active exercise and specific strengthening of the patient form also an integral part of physiotherapy whereas osteopathy is usually an exclusively passive intervention [17].

Nevertheless, overlaps and gray areas with other medical professions are reported. Not only physiotherapy but also chiropractic uses similar or partially identical treatment techniques:

*"[Which] are then also quite similar in the specifics, partly identical in the techniques." (O1, item 38)* [17]

The differentiation here is therefore not exclusively based on the precise execution of the individual techniques, but in particular on the various concepts and thought models of the respective discipline. In this context, the interviewed osteopaths repeatedly emphasized the holistic view of the human being as a central paradigm [17, 18].

#### Patient profile

All COCO interview studies show an extremely broad profile regarding osteopathic treatments. Patients of all ages are treated osteopathically.

*"Oh, everything actually, there are patients of all ages. From (…) three-month-old babies to over 90- year-old men, women, so I couldn't paint a typical picture." (O2, item 54)* [21]

Although diagnoses and leading symptoms from all medical disciplines are mentioned, most indications relate to conditions from the field of orthopaedics, such as back and neck pain, joint pain or scoliosis [18, 21].

Furthermore, headaches, migraines, tinnitus, temporomandibular joint problems, abdominal pain, hormonal imbalances, unfulfilled desire to have children or neurological conditions are also cited by the participants as indications for treatment [21]. The support of sport teams presents another area of osteopathy application [18].

The decision for osteopathic treatment and which osteopath the patient chooses depends on various factors. Recommendations from third parties, local availability or the therapist's specialization in a certain discipline or treatment method play an important role in the decision-making process.

*"Because the patients who come to me come by word of mouth, yes." (O5, item 47)* [21]

*"Everything else is, I think, very average, that is, all the people who come to me do so because I am in their vicinity." (O3, item 38)* [21]

What is the ultimate decisive factor remains an open question. However, one osteopath suspects:

*"When I see where my patients come from, {I come to the conclusion} that an established reputation is much more interesting than a location." (Interview 02, item 129)* [18]

In addition, professional training must be considered account for the acquisition of patients. In Austria, where osteopathy is not an independent profession, osteopathic treatment is usually prescribed by a physician. Therefore, one osteopathic physician acquired some of his patients directly from his conventional medical consultation hours [21].

#### Limits of osteopathy

There is general agreement that osteopathy has its limits. One osteopath consciously distances himself from the.

*"(…) image {idea} that almost everything can be treated with osteopathy (…)" (01, item 56)* [19].

This belief is hardly acceptable to him and he argues for clear limits regarding the treatment method. [19]*.*

One can additionally detect a consensus regarding general contraindications to osteopathic treatment, such as the primary treatment of a structural injury (e.g., bone fracture) or further medical emergencies [21]. To ensure patient safety in these cases, the diagnosis and treatment must be carried out by a physician [17, 18]. Moreover, osteopaths should not leave their area of expertise when advising. This, in particular, applies to medical interventions or vaccinations (O4, item 75) [21]*.*

Nevertheless, according to some participants, some diseases can be treated osteopathically at the same time or at a later stage.

*"Yes, of course I see the limits in the pathologies that are there. If there is actually (…) an osteoarthritis that is simply there and will not vanish, osteopathy shall certainly have its limits; one can perhaps relieve the pain, but the osteoarthritis cannot be cured by osteopathy, now it can. I also see limits in some diseases." (O7, item 56)* [21]

A clear objective and clear communication are crucial for the possible co-treatment of these diseases:

*"Well, that depends on the objective. (…) It all depends totally on the objective. If I say that I want to treat coxarthrosis curatively, I think that we shall soon reach our limits with osteopathy; if we do a control X-ray after six months, we shall see that it is still coxarthrosis. […] But if I say that I want to improve the quality of life, I would treat them nevertheless. The question is what the objective is." (O9, item 41)* [21]

Other respondents express concerns about the use of specific techniques (e.g., intravaginal and rectal techniques) and exclude them for legal reasons [17, 18].

#### Anchor figures

So-called anchor figures seem to play an important role in osteopathy. In many of the respondents' statements, one can find repeated references to formative personalities in osteopathy. Both historically influential and currently practicing as well as teaching osteopaths are mentioned here.

*"But there will always be people who really care about this innermost quality of osteopathy. (…) And just as osteopathy has developed from Still to Sutherland, Becker, Viola Frymann (…) and all their names (…), or Mitchell and Jim Jealous now (…), so it will continue to develop." (O5, item 84)* [21]

Osteopathy has been confronted with its various influences and diverse views of its founders and their successors since the very beginning.

*"[T]his separation between the philosophy and the intuitive part of osteopathy, and the rational part or the biomechanical part, was there right from the start (…) separation between Littlejohn and Still, and I think at the foundation of osteopathy, there has already been that argument and that polarization." (O5, item 37)* [17]

Participants feel that anchor figures polarize the community of osteopathic practitioners in education and training. Different streams and schools of thought are established through which osteopaths have to navigate and find their individual therapeutic path.

*"I also believe that the challenge among osteopaths is to reconcile the different styles and different directions or to develop the acceptance that it is so multi-faceted" (O4, item 99)* [19]

### Challenges of osteopathy

#### Identity problem

Many interviewees define their professional activities through historically conveyed treatment principles and models of thought. However, there is great disagreement among the respondents about the importance of these principles [17]. It seems that the "average" osteopath or "typical" osteopathic treatment does not exist.

*"[T]here's as many osteopathies as there are osteopaths. And probably even with one osteopath, there's as many types of cares, number of patients, so it's really hard labeling things." (O5, item 61)* [17]

The different training institutions and their curricula influence the inhomogeneous mass of osteopaths, which, in turn, affects intra-professional communication. Different terms are used for osteopathic diagnoses depending on the training institution, for example [17].

*"What you are told always depends on the training. The quality of a training course depends on the quality of the managers from this training institute and they come from a wide variety of backgrounds." (Interview 01, item 32)* [18]

The osteopath's previous professional training also has a decisive effect on the actual practice of osteopathy. For instance, osteopathic trained physicians act differently than “*physiotherapeutic osteopaths*” (O4, item 27) [18]. This manifests, for example, in conflict management.

*"Yes, of course I know that. I have a bonus, because I'm simply a doctor. And of course, osteopaths that aren't doctors have greater difficulties and are often rejected, (…) well, (…) because they are no medical doctors in a manner of speaking and (…) there are obviously difficulties." (O1, item 101)* [21]

Although patients can benefit theoretically from the different approaches of individual osteopaths, they may not know what to expect before an osteopathic intervention (O2, item 20) [19]. In the end, it remains questionable whether or how the patient can find a suitable osteopath.

The identity problem of osteopaths is probably the most fundamental challenge of the work field. The lack of clear definitions of the work not only affects the profession itself, but also its public image.

*"I would say that the identity problem is the most important issue. (…) The definition is the most difficult question and no one can answer that. And if we can't answer that and don't deal with it, how can we argue what we are if we ourselves don't know exactly what we are." (O6, item 49)* [21]

#### Disagreements within the osteopathic community

The search for the core professional identity in the field of osteopathy additionally unlocks conflict potential. Specific conflicts between individual streams of osteopathy are described within the osteopathic community. In Austria, osteopaths working “structurally” in contrast to those working “biodynamically” are partially at odds*:*

*"Yes, there is really a gap between biodynamic osteopathy and structural osteopathy." (O7, item 104)* [21]

In this context, one participant provides disagreement within the osteopathic community as one reason for the central obstacle to the desired legal regulation of the profession.

*"The problem concerning regulation is—and that's simply the case now and that's also the elephant in the room about which no one is talking—(…) the problem with regulation has always been cranio. (…) You cannot say it openly, but it was always the problem of craniosacral therapy, no matter who I talked to." (O9, item 93)* [21]

The lack of dialogue between the various specialized parties has not only a (negative) impact on professional policy, but it can also hinder the further development of the profession on a long term and thus possibly result in worse patient care.

*"I think the fear is more the lack of dialogue. Not being able to actually sit down and try and speak in the interest of patients. So I think my fear would be isolation. Isolation of the practitioners, isolation of the profession, so not hearing, what others / not hearing critics from other places and so not building up into better practitioners." (O5, item 27)* [17]

#### Education

With the exception of Switzerland, the training modalities in the German-speaking countries are anything but consistent:

*"The training is totally different" (O3, item 19)* [19]

Training to become an osteopath is either part-time or full-time. They usually last 4–5 years, depending on the degree one aims to achieve.

*"If we are talking about professionalization and standardization of vocational training / -image, about consistency, then we must also have a consistent curriculum." (Interview 02, item 171)* [18]

It is worth noting that this lengthy and extensive training may *not* qualify the student to practice osteopathy independently, even after successfully completing osteopathic training.

*"But what has also happened at the moment and fundamentally, consensus groups have also considered that an osteopath who does a four-year course […] is not qualified to work on a patient. […]. If I study agriculture or whatever, the bachelor's degree makes me employable and that's currently [not] the case in osteopathy (…).” (Interview 06, item 101)* [18]

In the course of the advancing academization of allied health professions, it is possible to acquire a bachelor's or master's degree in osteopathy. This serves as a basis for research in osteopathy and is well received by the osteopathic community.

*"I think it's good because I believe that academization leads to more being done in the field of science." (Interview 04, item 132)* [18]

To be able to transfer new findings from research into osteopathic practice, among other things, one participant pleads for the promotion of core competencies of scientific work to be taught right from the start of osteopathic training.

*"(Oh my) The training. (...) We should learn from the beginning, not only during the last year when we have to write a master thesis, we should learn from the beginning what it means to work in an evidence-based way, to do research (...). It works in physiotherapy and is continuously getting better there, but in osteopathy (...). At the beginning, we never learn to deal with available studies, it is a matter of training. From the beginning, not only during the fifth year shortly before the master thesis, we should have the first lessons in statistics." (O4, item 113)* [21]

However, critical voices among osteopaths regarding academization are also raised. For example, calls for a distinction between osteopathic practitioners and researchers are made. The integration of scientific learning content into teaching could even have a negative impact on the osteopath's technical skills.

*"What really defines us is that we treat patients. And just because you've studied full-time for five years and are academically qualified doesn't make you a good osteopath, that's for sure. Just because you know it in theory doesn't mean you can feel it, no." (Interview 03, item 133)* [18]

With the exception of Switzerland, no standardized training modalities in the German-speaking countries exist. As the profession of osteopath was only enshrined in law in 2020, one can currently find osteopaths who completed their training abroad or before regulation among the practitioners.

*"On the one hand, we only have one school in Switzerland and it's in Fribourg, the University of Applied Sciences. Ideally, I think it produces 25 new osteopaths per year." (O7, item 64)* [17]

Not only the topographical location of the sole training centers is considered problematic for German-speaking Swiss: The predominant language there, French, could present an obstacle for German-speaking Swiss.

*"I would like to see a university of applied sciences in German-speaking Switzerland." (O6, item 124)* [17]

#### 3.2.4. Research

Most osteopaths surveyed argue in favor of the advancement of scientific work in osteopathy and regard this as a great opportunity to establish the profession.

*"if you want to be taken seriously, then I think you have to do research, and in a society like ours you have to be able to put up with someone else doing research and asking questions about what you do." (Interview 05, item 153)* [18]

Moreover, training standards play a central role in the discussion about scientific work in osteopathy, as explained in the previous chapter.

*"I only think that if we also have people who do research professionally as professional researchers and osteopaths at the same time, that we can then produce academic literature of the quality that we don't have to hide from other therapeutic directions." (O2, item 75)* [19]

However, scientifically based research efforts are discussed with reservations. As a result, some parts of osteopathy that cannot be scientifically proven could be excluded from regulation on the basis of study results. Hence, some participants fear that the core of osteopathy could be lost.

*"If one tries now to take this out of this mental [Original German word: geistigen] (…) source, (…) I see the risk that it is practically shifted into evidence-based, as important as that is, well, but only into evidence-based, visible and perceptible dimensions, then osteopathy shall lose its soul from my point of view. (…) And that's actually the greatest threat to osteopathy for me." (O5, item 64)* [21]

Osteopathy is thus faced with the difficult task of advancing osteopathic research without sacrificing its principles.

*"A good scientific basis in order to argue how (…) many benefits osteopathy has (…)—in the end, (…) academization probably cannot be avoided and will certainly be necessary. Even if all these developments are not entirely without risks. That is, the risk of losing sight of the holistic aspects of osteopathy." (O1, item 81)* [21]

Furthermore, differentiated criticism of the study designs commonly used in manual therapy research is voiced and their applicability to osteopathy is questioned:

*"It doesn't make osteopathic sense to assess a single technique because every person is different and has accumulated different problems and compensations over their lifetime. In other words, you have to look individually and therefore you can't investigate whether individual techniques now help for an exact problem, that is precisely not the osteopathic way of thinking." (O2, item 72)* [17]

Many of the interviewees describe paradigms and concepts as the core of osteopathy. Investigating the effect of a specific treatment technique is not central to them. This raises the question of what a research design could look like.

*It is difficult to reconcile the way we think with today's scientific criteria." (O2, item 72)* [17]

In the opinion of one participant, systematic basic research seems to be needed to identify what characterizes osteopathy before investigating its effectiveness.

*"So, I think qualitative study is something very important now, because we have a lack of grounding, so for exploring things, we need to go through that." (O5, item 41)* [17]

In the opinion of one of the osteopath interviewed, it is not only the war of faith that impedes the progress of osteopathic research, but also high costs and a lack of staff to carry out large-scale studies are additionally perceived as obstacles (O2, paragraph 78) [17].

Despite the reservations expressed, some of the interviewed osteopaths are aware that it also requires questioning and relinquishing of old beliefs to advance with the treatment method.

*"I do think it requires a lot of rethinking and letting go on certain false beliefs we've had." (O5, item 33)* [17]

#### Country-specific professional situation: Germany

In Germany, physicians, naturopaths and physiotherapists work in the field of osteopathy. According to a federal court of 2015, the unrestricted practice of osteopathy is reserved exclusively for physicians and naturopaths. Osteopaths with prior training in physiotherapy are presenting a large proportion of practitioners in Germany. They are required to take an exam to become a naturopath to be able to practice osteopathy with legal certainty [7]. This classification of osteopathy by the supreme court is viewed differently by the participants [18]. It raises the question of whether and how exam-relevant content should be integrated into osteopathic training to become a naturopath. The participants criticize the current professional legal situation not only because of the lack of a prescribed training as a naturopath:

*"And logically, you always have to ask yourself (…) what is the quality of training as a naturopath (…) and to what extent should this be a basic requirement in order to then have the right to treat independently as an osteopath."* (Interview 01, item 241) [18]

*"I think it's a scandal that trained osteopaths have to take a naturopath exam (…) to get a work permit. I find that scandalous."* (Interview 04, item 118–119) [18]

However, some participants, who are licensed as naturopaths, also see the current professional policy situation as highly positive as it offers osteopaths, who are not trained physicians, great freedom, such as the practice of osteopathy "first contact". As long as osteopathy is not yet regulated as an independent profession, the interviewees consider the practice of osteopathy for non-physician osteopaths to be a solid interim solution [18, 19].

*"But as long as osteopathy is not an independent profession, that is the best way." (Interview 08, item 167–168)* [18]

Patients perceive the unclear professional situation of osteopathic practitioners as opaque. [19] It is not clear to outsiders what qualifications the consulted osteopath has. A risk of less qualified therapists, who have not completed the 4-year training recommended by osteopathic organizations and who us the term “osteopath”, which is unprotected in Germany, can be identified [18].

*“[There is a danger] that some bums with a few weekend courses can call themselves "osteopaths"" (O1, item 64)* [19].

Finally, fears of a legal gray zone, which could arise in the event of a possible abolition of the naturopath profession or another legal regulation, in the practice of osteopathy exist [18]. Ultimately, the desire for the establishment of an independent profession is expressed repeatedly. Nevertheless, concerns and worries are expressed also in response to the question of a separate occupational profile. For example, some participants fear disadvantages as a result of an occupational law, such as financial losses or restrictions in the practice itself.

*"(…) (thinking about it) (…) I have mixed feelings about it, for example in England osteopathy is a profession and because it is a profession, they have restrictions on what they can and cannot treat and that is no longer osteopathy" (Interview 08, item 145–147)* [18]

The large number of professional organizations and their work is also criticized by the respondents [18].

*“(…) it's always about politics. Well, it seems that therapists are always arguing. I don't know how many therapy associations there are, just in one country, not to mention many other countries. It seems to be in the DNA of therapists.”* (Interview 09, item 39) [18]

#### Country-specific professional situation: Austria

Despite a lack of legal regulation of osteopathy practice in Austria, with physiotherapists on the one hand and physicians on the other, the professional landscape is much clearer—without naturopaths, because the category of naturopaths does not exist here.

The physicians practice osteopathy largely without restrictions.

*"No, I'm a doctor, I have (…) no restrictions." (O9, item 85)* [21]

Osteopaths who have completed physiotherapy training, depend on prescriptions from physicians. This significantly limits them from practicing osteopathy. It is reported that some osteopathically trained physiotherapists practice osteopathy on physiotherapeutic prescription slips.

*"Many colleagues work as physiotherapists, they also charge for osteopathy as physiotherapy, and yes, they are refunded in this way. And as a result, (…) osteopathy is also a little (…) less in the focus than it should be. I've been working for 20 years now, I'm only writing osteopathic invoices. (…) But (…) of course (…) I understand the problem. If somebody has fewer patients (…) and (…) has to charge for (…) physiotherapy, I absolutely understand the situation. (…). But (…) these problems are of course (…) long-burning issues." (O5, item 78)* [21]

This approach also means that treatment costs can be covered by health insurance, which would otherwise only be possible with an additional insurance for osteopathic therapy.

*"No, [the bill] of course says physiotherapy and remedial massage, because otherwise the patient doesn't get his/her money from the insurance company. For the insurance company, well, this is ok or it is tolerated. I have already received the feedback from many patients that they told the company that they went to an osteopath, and the health insurance said that of course that can't be billed, but we shall write physiotherapy and remedial massage and then that's it." (O8, item 55)* [21]

#### Country-specific professional situation: Switzerland

Among the German-speaking countries in Europe, Switzerland occupies a special position due to the creation of the independent profession of osteopathy and its implementation in the healthcare system.

*"[I]n Switzerland in particular, the entire training program has now really been regulated. It has a training qualification that is defined and I think we have come a long way, or further, throughout Europe."* (O1, item 48) [17].

However, even after the successful legal regulation of osteopathy, uncertainties among Swiss osteopathy practitioners are rampant.

*"So here in Switzerland, this, this implementation of the new Health Professions Act will certainly be a big challenge at first. Especially in German-speaking Switzerland with the integration of all these osteopaths who don't have a GDK title." (O4, item 95)* [17]

Whereas osteopaths with part-time and full-time training from Switzerland and abroad were previously able to practice osteopathy, osteopaths with the Swiss GDK–CDS diploma (dt. Konferenz der kantonalen Gesundheitsdirektorinnen und -direktoren/en. Swiss Conference of Cantonal Health Directors) have a clear advantage under the new legal regulation.

*"So those [osteopaths] who have the Swiss diploma are doing well, because they are "cleaned and groomed", they can do what they want." (O3, paragraph 60))* [17].

Osteopaths whose osteopathic training does not meet the current requirements of the newly created professional law can no longer practice under their own professional responsibility. However, there are also gray areas that allow for practicing without further training or re-examination. For example, the therapists in question can be employed by osteopaths with a GDK–CDS diploma or they can continue to practice as physiotherapists [17].

*"Other colleagues didn't have that. They simply carried on practicing osteopathy in their practice. Sometimes that was okay, sometimes some were reprimanded, i.e. warned, legally, with fines, which is still the case today." (O7, item 16)* [17].

For the participants negatively affected by these regulations, this also results in existential fears (O7, item 113) [17].

### Opportunities of osteopathy

The future of osteopathy is viewed highly as positive across all countries [17–19]. The participants report a very fulfilling daily practice routine and therapeutic successes [17, 18] with many positive patient responses [19].

*"As the coolest [profession] in the world."* (Interview 04, item 47) [18]

This may also be explained by the fact that, in countries without a legal regulation of osteopathy, one’s own work can be organized more freely.

*"(thinking about it) honestly quite good, because they [osteopaths] are in a gray area and are still um for example when it comes to pricing um actually allowed to call for what they would like to have. They are also free to set the times they want to use and they are also free to decide how and which techniques they want to use. So that's quite a / um quite a luxury situation, in my opinion."* (Interview 02, item 137) [18]

Furthermore, the interviewees almost consistently reported an extremely high demand for osteopathy from the population [17–19].

Especially, in Germany and Austria, a professional development of the osteopathic profession is seen as a great opportunity, as almost all respondents admitted [21]. As more and more neighboring countries are enshrining osteopathy in law and regulating training and association structures in a binding manner, professional recognition in Austria and Germany now also appears to be within the realm of possibility [18].

In addition, the osteopaths surveyed see the possibility of extending the field of osteopathy to areas such as disease prevention or integration into the palliative care of patients [21]. Moreover, research in the field of osteopathy is not only seen as a challenge but also as a great opportunity to establish the treatment method.

*"I think we are only at the beginning."* (Interview 08, item 210) [18]

### COVID-19

The COVID pandemic, which began in March 2020, had a significant impact on the healthcare systems of all the countries in question in the studies included. However, not all of the studies analyzed describe the effects of COVID-19 on the practice of osteopathy. One of the studies was conducted before the COVID pandemic [17]. COVID is not mentioned in the presentation of the results of another study [19]. Bücker quotes test subjects with existential fears at the beginning of the COVID pandemic [18]. Manschel et al., whose data was collected during of the pandemic, describe not only the financial losses osteopaths suffered from but also the restrictions in communication between patient and therapist, the change in the clinical images of their patients and the diverse effects on osteopathic teaching as well as on further and advanced training [21].

## Discussion

### Characteristics of osteopathy

Many of the respondents had difficulties with verbalizing osteopathy and its characteristics. According to the analysis of how the osteopaths surveyed describe osteopathy themselves, it is noticeable that there is indeed a certain degree of agreement between the characteristics they mentioned and the official WHO standards [1]. Nevertheless, many participants find it difficult to precisely define osteopathy and its scope of practice. This study cannot conclusively clarify the reasons for this uncertainty among the professional group. However, it can be assumed that the majority of osteopathic practitioners is inconsistent and that the different legal situations in the various countries described above as well as questions of prior professional training and, eventually, conflicts between the various streams within osteopathy may have played a role [17–19, 21]. Comparisons with other similar health care professions were used to emphasize the professional differences. This has also been the case for the results of other studies as well [24].

According to the interviewees, a central quality of osteopathy is the generous consultation time and the individual approach to the patient. Its effect was explained by a participant as being placebo, which is not tenable in a scientific context. Psychosomatic effects and "non-specific" effects (e.g., touch, relationship, cognition, agile approach to the understood needs of the patients) play a major role in the effect of the treatment, which is often erroneously referred to as a placebo [25]. The specific osteopathic palpation with its findings is also emphasized by the osteopaths surveyed as a special quality. It is worth mentioning here that palpatory findings are often not radiologically detectable and, therefore, the need for other verification, including peer palpation, must be considered.

This study also showed that the respondents' answers often referred to the statements of anchor figures. These anchor figures polarize osteopathic practitioners in education and training. Different streams and schools of thought are established through which osteopaths have to navigate to find their individual therapeutic path. The possible influence of anchor figures is discussed in depth in relation to training and research in the challenges of osteopathy in the next chapter.

### Challenges of osteopathy

The participants own professional understandings in contrast to other medical professions and their anchoring in osteopathic values or philosophy are similarly described in other studies on professional identity [24]. Research osteopathic professional identity is about progressing internationally. As Phillips points out in her review, the scope for diversity in osteopathy is determined by the legal framework and the deep-seated tensions challenge the perception of our collective identity [26].

The OPERA project has surveyed the professional identity of osteopaths in several European countries. It is noteworthy that in Austria, with 17% of the respondents presenting the lowest percentage of the countries surveyed, professionals see themselves exclusively as osteopaths [11]. In contrast, in a survey of German osteopaths, 93.62% identified strongly as osteopaths [9], despite the fact that Germany has no professional law either. Looking at the results of the study, the different professional backgrounds of osteopaths are shown to have an impact on their own understanding of the profession. For example, (non-physicians) osteopaths and osteopathic physicians in Germany and Austria face different challenges. In his survey of German osteopaths, Dornieden also recognizes the differences between practitioners in terms of their professional backgrounds, professional views and goals. He considers these factors as crucial for the evolvement of diversity within the community of osteopathic practitioners [9]. It seems reasonable to suggest that this divergence in qualification prior to osteopathic training essentially also applies to Switzerland. Unfortunately, there were no medical osteopaths in the Swiss COCO partial study.

Conflicts within the osteopathic community are reported in all of the studies analyzed [17–19, 21]. A division of osteopathic practitioners into different camps was only explicitly described in Austria. Here, the differences between the therapeutic approaches of ‘structural’ and ‘biodynamic’ osteopaths were described by participants as having a major impact on the professional image. [21]. The twofold division of the professional group of osteopaths into two camps regarding the use of a particular treatment method has also been described in other countries [3]. As this is not apparent in Germany or Switzerland in the present data set, it can be regarded as either a country-specific phenomenon or it was not discussed in sufficient depth in the interviews. In addition, the age of the osteopaths can possibly influence the use of certain techniques. In a survey in Switzerland, a higher age of the osteopaths interviewed was positively associated with the use of biodynamic technique [14].

These intraprofessional conflicts are not specific to osteopathy, but can also be found in the chiropractic profession [3, 27, 28]. Similar to our results, in a survey of chiropractors from Germany (who are also interested in creating their own professional profile), respondents argued that scientific work could create acceptance for the profession [29]. A clear presentation is also important for a later integration into the healthcare system. A study in Australia has shown that still large gaps exists in insurers' knowledge about what can be done well with osteopathy [30].

The question arises: what skills and abilities should osteopathic training teach to the osteopath Indeed, without standardized training, this is difficult to answer and practitioners are well aware of these grievances?. The question of what and how should be taught is an intensely debated one in the field that, unfortunately, has hardly been investigated empirically [31]. Expanding the curricula might positively influence professional identity and the perception of the profession. Esteves et. al argue in favor of implementing the concept of person-centered care in osteopathy [32]. A French study points out that more research regarding person-centered care in osteopathy is needed to meet the needs of patients, osteopaths and teachers [33]. In Germany, for example, there are repeated efforts by various professional associations to commit themselves to qualifications [34] and to appeal to internationally valid standards [1]. But even after regulation, as in Switzerland, the question remains of how to deal with osteopaths who have already completed a training that does not comply with the regulations. Notwithstanding in Switzerland, osteopaths with a GDK–CDS diploma and osteopaths with unrecognized qualifications face completely different challenges. There are several court rulings regarding the recognition of osteopathic training qualifications in Switzerland [35, 36]. The recognition of qualifications from abroad is particularly difficult if osteopathy is not recognized as a profession in the country of training itself [37]. In addition to the special recognition of foreign diplomas (e.g., Italian or French), Switzerland offers applicants the opportunity to take exams, even if the profession is recognized in the country of origin. These differences refer to the professional training in Germany and Austria, where osteopathy is not yet regulated as an independent profession. In the meantime, the degrees from neighboring countries are also checked for approval [38], but this also shows the importance of considering osteopathy as a whole in German-speaking countries. The regulation of a profession may also affect neighboring countries. Cerritelli et al. argue for an European harmonization of curricula in Europe to not impair the mobility of osteopaths working in neighboring countries [39]. Especially without the language barrier, this is of great importance for the German-speaking area with commuters in border regions.

However, the situation in Switzerland also clarifies that the establishment of an independent profession is not the ultimate solution to the problems mentioned above. The diversity of the osteopathic community with its various educational backgrounds, professional training and its specific cultural approaches to osteopathy seems to lead to major differences in practice even after regulation. A study from 2018 has already identified challenges for Swiss osteopathy regarding future needs for professional training, standards and quality of care with professionals working in isolation, quality of record keeping and consent as well as shared decision making with patients. However, the survey may reflect the situation of osteopaths in Switzerland at a point in time before the establishment of the independent profession [40]. In Germany, the profession of naturopaths presents a special case. If non-physician osteopaths pass an exam of naturopath, they can practice without being bound to a physician's referral. At first glance, this possibility could present a clear advantage to the (non-)regulation in Austria. Ultimately, this solution may be part of the problem or could even become an obstacle to the establishment of a new profession, though. Since non-physician osteopaths currently can practice in a legally secure manner, a need for legal regulation does not seem urgent. The situation is difficult and can hardly be grasped by other medical professions. According to a study, general practitioners in Germany could also benefit in their function as health-care adviser from clear information about osteopathy, including the legal situation of the practitioners [41].

In terms of training standards, scientific research should also be mentioned. In general, the academization of therapy professions advances. For example, according to a questionnaire study from 2022, more than one third of the osteopaths surveyed in Austria have a master's degree [11]. However, it is questionable how many of these osteopaths continue to work in research after graduation. A look at the studies reveals much subjective skepticism about the scientific approach to osteopathy. In the course of an article on osteopathic empirical research, a bibliometric analysis was carried out from 1966 to 2018. Looking at the countries of the evaluated publications, it is striking that only 3.1% of the total publications are from Germany and 1% from Austria [42]. Repeatedly, participants fear that osteopathy could lose its "true self" in the process. How should the ambivalence of the osteopaths interviewed on the subject of research be dealt with? It is not new to the osteopathic community that osteopathy has to critically question old paradigms, as for example Thompson et MacMillan have called for, not at least for the safety of the patient [43]. From the authors' point of views, research must always adapt to the subject and not vice versa. This applies not only to research in osteopathy or medicine, but also to other fields of research [44]. Morin and Gaboury, for example, call for research designs to be designed in such a way that osteopathy can be studied in detail, such as individual case studies [42]. There may also be opposing economic interests that research complicate. In Germany, for example, there is a great demand for complementary and alternative medicine from patients, but this is not universally accepted in the scientific community [45]. In this context, the regulation of the profession and its integration at universities remains relevant for osteopathy too. Leading in osteopathic research are countries with well-organized regulation of the profession and university education, such as in the United States, Australia and the United Kingdom [42]. The influence of teaching anchor figures probably also plays a major role here. For example, it is initially difficult for osteopathic trainees to distinguish between teaching content based on evidence-based research or on personal experience or personal opinion of the lecturer. In addition, pseudoscientific claims could threaten the credibility of osteopathy as healthcare profession [46]. It is also interesting to note that most of the international osteopathic literature is published primarily in osteopathic journals, which complicates interdisciplinary work [42]. Several studies in neighboring European countries explore the integration of research findings into the daily practice of osteopaths. The willingness of osteopaths to transfer evidence-based practice (EBP) into their daily work with patients has already been subject to research in other European countries [47, 48]. Among practicing osteopaths, there seems to be an openness to evidence-based practice (EBP) at the transnational level, but the ability to handle the former varies from country to country. A study from Spain shows that inadequate legal regulation of the profession and training could be linked to osteopaths' inadequate scientific work skills [49]. Especially in countries without state regulation of osteopathy, the qualification of practitioners is difficult to assess. In their 2022 survey, van Dun et al. showed that a quarter of Austrian osteopathic practitioners surveyed could not answer which osteopathic qualification they held [11]. How the regulation of the profession affects the scientific work skills of osteopaths in German-speaking countries has yet to be investigated.

### Opportunities of osteopathy

The satisfaction of osteopaths with their work and the high demand for osteopathy in society are described as opportunities for the discipline. According to a Forsa survey in 2024, more than 19 million people in Germany will have received osteopathic treatment [50]. The demand for osteopathic treatment seems to be there and the profession has reached the mainstream. Patients also seem to be satisfied with the treatment; quantitative studies have shown that more patients are satisfied with osteopathy than are not [51].In Germany and Austria, most interviewees saw the creation of a well-defined profession as an opportunity for osteopathy. The effects of (non-)regulation on professional identity, education and research, among other things, have already been discussed in detail in the previous sections. What should be treated with osteopathy and how also depends on the design of professional laws. It is possible to extend the scope of practice to include prevention, as suggested by one osteopath surveyed. Alternatively, as in Italy, the professional profile could be based on prevention, with all its advantages and disadvantages [39].

### COVID-19

The effects of the COVID-19 pandemic on osteopathic practice described here are underpinned by initial studies that show a particularly large impact of the pandemic on training activities [52]. The impact of the pandemic on the practice of osteopathy in Switzerland in 2020 was the subject of a survey. According to the survey, 66.3% of the osteopaths surveyed were unable to work for a short period of time due to measures to mitigate the pandemic. According to the authors, the financial losses osteopaths suffered from can be compared to those of parental leave [53]. Further studies must show whether this can also be transferred to the German-speaking countries.

## Strengths and limitations

First, it is indispensable to note that the results of the metasynthesis do not necessarily allow conclusions for the whole population of osteopaths in the countries in question as this is not an evaluation of representative surveys with large case numbers. Nevertheless, tendencies seem to emerge when osteopaths' statements from the various studies appear to be congruent or confirm or complement each other in a meaningful way.

The size of the data set definitely presents a limitation of the study. Although studies from all three countries were available and a total of 30 expert interviews was analyzed, the sample could be considered as an absolute lower limit for a metasynthesis. In a study by Hennink and Kaiser, 23 articles were identified that used empirical data (*n* = 17) or statistical modeling (*n* = 6) to assess saturation. The result of the study was that studies that used empirical data achieved saturation within a range of interviews (9–17) [54].

With regard to the data basis for the synthesis, the literature research must also be included. The research was done with the aim of identifying new studies. Unfortunately, in the end only the COCO study itself could be used. One could argue that the exclusion criteria were formulated too narrowly. However, the overriding goal was to only find qualitative studies on the research questions of the COCO project. In summary, it can be said that little has been published on this topic so far and that this work can provide the basis for further research.

Whether the results of this synthesis can also be transferred to the entirety of osteopaths in German-speaking countries will be investigated by means of a representative survey. The COCO study protocol therefore envisages a transnational quantitative questionnaire study as next step to test the general validity of the findings obtained so far.

## Conclusions

Conclusive characterization of osteopaths and their work in German-speaking countries still presents a challenge.

Regarding the characteristics of osteopathy in Germany, Austria and Switzerland, it can be said that osteopaths find it difficult to define osteopathy. When it comes to qualities, they particularly mention manual work on the patient, a holistic approach to treatment, and generous treatment durations. The patient profile is broadly diversified across all medical specialties. The specific osteopathic palpation is used to distinguish itself from other medical professional groups. The limits of osteopathic treatment are mentioned and put particularly into context with the respective objective of the treatment. Osteopathic lecturers and special (historical) personalities can be seen as anchor figures and influence the view of osteopaths.

If one aims to identify the challenges, the identity problem of osteopaths is central. In addition, there are differences in training and qualifications depending on the country and school. The need for scientific research is perceived differently. To conclude, osteopathy in German-speaking countries faces major challenges. It must position itself, particularly in Germany and Austria, to avoid remaining in a gray area. The question of its own identity is central to this: what defines osteopathy today? What can and should osteopaths do for society and what not? Old thought patterns and paradigms do not necessarily have to be abandoned, but they must be systematically and critically questioned. Furthermore, the different professional policy situations must be considered in this context. In the case of further legal regulation of the profession or training, the effects on all osteopaths with their different professional qualifications should be reflected upon.

The satisfaction of osteopaths in their work and the extreme demand for osteopathy in society should regarded as an opportunity for the field as should be the expansion of the scope of practice can also be seen as an opportunity.

It remains remarkable that despite the different professional situations in the home countries of the interviewed osteopaths, the characteristics, challenges and opportunities of osteopaths hardly differ from each other.

## Supplementary Information

Additional file 1.

Additional file 2.

## Data Availability

No datasets were generated or analysed during the current study.
