# Supplementary material for: Characteristics, opportunities, and challenges of osteopathy (COCO) in the perceptions of osteopaths in Germany, Austria, and Switzerland: a metasynthesis
Source: Eur J Med Res. 2024 Dec 18;29:586. doi: 10.1186/s40001-024-02199-3 (PMC11658149; doi:10.1186/s40001-024-02199-3)
Supplement: Supplementary file 1 — Additional file 1. [file 40001_2024_2199_MOESM1_ESM.docx]

**Table S1**: Bibliographic Databases Search

| Database | Search-String | Filter | Date | Hits |
| --- | --- | --- | --- | --- |
| Pubmed | (osteopath*) AND (german* OR austria* OR swiss OR switzerland) AND (characteristics OR profession OR training OR attitudes OR beliefs OR identity OR definition OR profile) NOT (clinical OR metabolic OR tissue OR animal OR vaccination OR oncology OR trauma) | Publication  < 10 years | 23.3.2023 | 40 |
| CINAHL | (osteopath*) AND (german* OR austria* OR swiss OR switzerland) AND (characteristics OR profession OR training OR attitudes OR beliefs OR identity OR definition OR profile) NOT (clinical OR metabolic OR tissue OR animal OR vaccination OR oncology OR trauma) | Publication  2013-2023 | 23.3.2023 | 5 |
| PSYCINFO  (via EBSCOhost) | (osteopath*) AND (german* OR austria* OR swiss OR switzerland) AND (characteristics OR profession OR training OR attitudes OR beliefs OR identity OR definition OR profile) NOT (clinical OR metabolic OR tissue OR animal OR vaccination OR oncology OR trauma) | Publication  2013-2021 | 23.3.2023 | 8 |
| Cochrane Reviews | osteopath* NOT intervention | "title abstract keyword" | 23.3.2023 | 1 |
| PEDro | osteopath*, characteristics | Publication  > 2013 | 16.3.2023 | 6 |
| OSTLIB | osteopath NOT effective NOT dysfunction AND german OR austria OR swiss OR switzerland | all studies and articles,  < 10 years* | 25.3.2023 | 98^1^ |
| total | | | | 158 |
| duplets | | | | 14 |
| total without duplets | | | | 144 |

^1^The filter "publication date" did not work. The search resulted in a hit count of 198. If the studies older than 10 years (since 2013) are manually excluded, 98 remained.

. **Table S2:** Reviewed Literature

|  |  | Reason for exclusion JM (Titel) | Reason for exclusion JM (Abstract) | Reason for exclusion JM (Fulltext) | Reason for exclusion JP (Titel) | Reason for exclusion JP (Abstract) | Reason for exclusion JP (Fulltext) |  |
| --- | --- | --- | --- | --- | --- | --- | --- | --- |
| Author (Year) | **Included** |  |  |  |  |  |  | **Title** |
| Engemann et Franke (2023). |  | Interview. |  |  |  | No qualitativ Study |  | In dialog with... Helge Franke. |
| MacMillan et Draper-Rodi (2023). |  | Scoping-review. |  |  | Protocoll |  |  | Osteopathic education: A scoping review protocol. |
| Barnes et al. (2022). |  | Effectiveness of OMT. |  |  |  | Clinical Study |  | Retention of tissue texture change after cervical muscle energy and high velocity low amplitude intervention: implications for treatment intervals. |
| Brandl et al. (2022) [1]. |  | Effectiveness of OMT. |  |  | Cohort Study |  |  | Influence of Rolfing Structural Integration on Active Range of Motion: A Retrospective Cohort Study. |
| Brandl et al. (2022) [2]. |  | Not primarily osteopathic. |  |  |  | No qualitativ Study |  | Associations between Deformation of the Thoracolumbar Fascia and Activation of the Erector Spinae and Multifidus Muscle in Patients with Acute Low Back Pain and Healthy Controls: A Matched Pair Case-Control Study. |
| Chen et al. (2022). |  | Physiotherapy study. |  |  |  | No qualitativ Study |  | Chest physiotherapy for pneumonia in adults. |
| Colati et Pagano (2022). |  | Effectiveness of OMT. |  |  | Case study |  |  | Effectiveness of Osteopathic Treatment on the Spinal Column as Measured by the Spinal Mouse®: A Case Series. |
| Cooke et al. (2022). |  | Lab-Study. |  |  |  | No qualitativ Study |  | The CRW1 Index: Identification of Eyes with Previous Myopic Laser Vision Correction Using Only a Swept-Source OCT Biometer. |
| Engel (2022) [1]. |  |  |  | Different design. |  | No qualitativ Study |  | The history of osteopathy in Germany. |
| Engel (2022) [2]. |  |  |  | Different design. | Review |  |  | German-language osteopathic literature: a survey from the discovery of osteopathy to the year 2000. |
| Gassner et Hofer (2022). |  | Effectiveness of OMT. |  |  |  | No qualitativ Study |  | Osteopathy: effectiveness and safety for musculoskeletal pain and overview of training and quality requirements. AIHTA Project Report No.: 144 2022. |
| German et al. (2022). |  | Training of medical students. |  |  | No qualitativ Study |  |  | The Confidence and Accuracy of Medical Students Trained in Ultrasound or Landmarks for Performing Knee Aspiration. |
| Golz et al. (2022). |  |  | Not primarily osteopathic. |  |  | other topic |  | Preparing students to deal with the consequences of the workforce shortage among health professionals: a qualitative approach. |
| Mayerl et al. (2022). |  | Animal-Testing. |  |  | No qualitativ Study |  |  | Regional Variation in Contractile Patterns and Muscle Activity in Infant Pig Feeding. |
| Meier-Girard et al. (2022). |  |  | Not primarily osteopathic. |  |  | other topic |  | Prevalence, specific and non-specific determinants of complementary medicine use in Switzerland: Data from the 2017 Swiss Health Survey. |
| O'Donovan et al. (2022). |  | Osteopathy as disease. |  |  | No qualitativ Study |  |  | Associations of body mass index and sarcopenia with screen-detected mild cognitive impairment in older adults in Colombia. |
| Richter (2022). |  | Conference report. |  |  | No qualitativ Study |  |  | Online Conference: Osteopathy Meets Psychology. November 19-20, 2021. |
| Schleusener (2022). |  |  |  | Essay about the evidence of OMT. | No qualitativ Study |  |  | Osteopathic medicine - Where is the evidence? |
| Seddon et al. (2022). |  | Surgery Training. |  |  | other topic |  |  | Feasibility and Potential for Real-Time 3D Vitreoretinal Surgery Telementoring. |
| Stecker (2022). |  | Historical Impression. |  |  | other topic |  |  | Impressions of A.T. Stills osteopathy. |
| Tan et al. (2022). |  | Osteopathy as disease. |  |  | other topic |  |  |  |
| Terrell et. al (2022). |  | Effectiveness of OMT. |  |  |  | other topic |  | Effects of osteopathic manipulative treatment versus osteopathic cranial manipulative medicine on Parkinsonian gait. |
| van Dun et al. (2022). |  |  |  | Different design. |  | No qualitativ Study |  | The Austrian Osteopathic Practitioners Estimates and RAtes (OPERA): A cross-sectional survey. |
| Beer et al. (2021). |  |  | Different study design. |  |  | other topic |  | Use of osteopathic medicine for chronic and long-term patients. |
| Daake (2021). |  | Not primarily osteopathic. |  |  | Review |  |  | How does the health disorder of idiopathic asymmetry and positional plagiocephaly in infants in the first year of life relate to therapy? A systematic literature review. |
| Garrido-Cumbrera et al. (2021). |  | Not an osteopathic study. |  |  |  | other topic |  | Gender differences in patient journey to diagnosis and disease outcomes: results from the European Map of Axial Spondyloarthritis (EMAS). |
| Hides et al. (2021). |  | Not an osteopathic study. |  |  |  | other topic |  | The effects of exposure to microgravity and reconditioning of the lumbar multifidus and anterolateral abdominal muscles: implications for people with LBP. |
| Krause et al. (2021). |  | Physiotherapy Study. |  |  | Physiotherapy |  |  | Medical exercise and physiotherapy modes and frequency as predictors for a recurrence of chronic non-specific low back pain. |
| Lüthi et al. (2021). |  | Not primarily osteopathic. |  |  |  | other topic |  | Complementary and alternative medicine use by pediatric oncology patients before, during, and after treatment. |
| Pakanecz (2021). |  | Observational Study. |  |  |  | other topic |  | Performance of Listening-Tests in the Context of Routine Osteopathic Findings – A Video-Based Observational Study. |
| Schwerla et al. (2021). |  |  | Different study design. |  |  |  | other topic | Most common infant health concerns in osteopathic practices in Germany. A survey. |
| Stecker et Scheuchl (2021). |  |  |  | Not exactly matching our research question. |  |  | other topic | Science and research in osteopathy - Part 1: The concept of science in osteopathy. |
| Zalpour et al. (2021). |  | Physiotherapy study. |  |  | Physiotherapy |  |  | A physiotherapeutic approach to musicians' health—Data from 614 patients from a physiotherapy clinic for musicians (INAP/O). |
| Anderseck et al. (2020). |  |  | Physiotherapy study. |  |  | other topic |  | Der aktuelle Stand der Employability (Beschäftigungsfähigkeit) von OMT-Absolventen der muskuloskelettalen (manuellen) Therapie in Deutschland. |
| Bäumer (2020). |  | Childrens foot function. |  |  |  | other topic |  | Children's foot function and subsequent adult's postural health: Cause and effect - Two sides of a single coin. |
| Biberschick (2020). |  | Interview. |  |  |  | No qualitativ Study |  | In dialog with … Diana Stöckl. |
| Bill et al. (2020). |  |  | Different study design. |  |  |  | other topic | Osteopathy in the French-speaking part of Switzerland: Practitioners' profile and scope of back pain management. |
| Brintrup et al. (2020). |  |  |  | Not exactly matching our research question. |  |  | other topic | Brintrup et al. (2020): Osteopathy for newborns in hospitals?: A qualitative study. |
| Calderón-Garcidueñas et al. (2020). |  | Lab-Study. |  |  |  | other topic |  |  |
| Dräger et Heller (2020). |  |  | Different study design. |  |  | other topic |  | Osteopathy under scrutiny. |
| Engemann et Resch (2020). |  | Interview. |  |  |  | No qualitativ Study |  | In dialog with...Professor Karl-Ludwig Resch. |
| Franke (2020). |  | Commentary. |  |  |  | No qualitativ Study |  | Osteopathy for the treatment of non-specific back pain: Critical comments on the results of the IGeL-Monitor. |
| Meier (2020). | x |  |  |  |  |  |  | Characteristics, Opportunities and Challenges of Osteopathy in the Perception of Osteopaths in Switzerland - a Qualitative Study - the Complete Research Report. |
| Meier et Porthun (2020). |  |  |  | Complete research report already included. |  |  | complete research report already included | Characteristics, Opportunities and Challenges of Osteopathy in Switzerland. |
| Merkt et al. (2020). |  | Not an osteopathic study. |  |  | other topic |  |  | Medical emergency in the practice: I pack my suitcase … |
| Putschögl et Woisetschläger (2020). |  |  | Different study design. |  | other topic |  |  | Knowledge of Nutritional Issues among Osteopaths in Austria: A Cross-Sectional Study. |
| Rauch (2020). |  | Effectiveness of OMT. |  |  | other topic |  |  | Sternal Recoil as an alternative to thoracic HVLA? |
| Rotter et al. (2020). |  | Not primarily osteopathic. |  |  | other topic |  |  | Use of Complementary Medicine in Competitive Sports: Results of a Cross-Sectional Study. |
| Zegarra-Parodi et al. (2020). |  | Commentary. |  |  | other topic |  |  | Extension of the biopsychosocial model for musculoskeletal practice. Part 2: Clinical significance for musculoskeletal treatment. |
| Capriles et al. (2019). |  | Archaeological sites. |  |  |  | other topic |  | Persistent Early to Middle Holocene tropical foraging in southwestern Amazonia. |
| Dubois et al. (2019). |  |  | Different study design. |  |  | No qualitativ Study |  | Characteristics of complementary medicine therapists in Switzerland: A cross-sectional study. |
| Griessenauer et al. (2019). |  | Not an osteopathic study. |  |  | other topic |  |  | Pharmacy-Mediated Antiplatelet Management Protocol Compared to One-time Platelet Function Testing Prior to Pipeline Embolization of Cerebral Aneurysms: A Propensity Score-Matched Cohort Study. |
| Gugliucci et O'Neill (2019). |  | Not fitting into the study profile. |  |  | other topic |  |  | Health professions education: Advancing geriatrics and gerontology competencies through Age-Friendly University (AFU) Principles. |
| Klumpp (2019). |  | Physiological phenomenon. |  |  |  | other topic |  | Heart rate variability in manual medicine. |
| Kraml (2019). |  | Not fitting into the study profile. |  |  | other topic |  |  | The role of mental imagery in osteopathic palpation. |
| Porthun et Manschel (2019). |  | Study-Protocol. |  |  |  | Not a Study |  | Characteristics, Opportunities, and Challenges of Osteopathy (COCO) in the Perceptions of Osteopaths in Germany, Austria, and Switzerland: Protocol for a Comprehensive Mixed Methods Study. |
| Putschögl (2019). |  | Not fitting into the study profile. |  |  | other topic |  |  | Knowledge of Nutrition Issues among Osteopaths. |
| Rehak (2019). |  | Effectiveness of OMT. |  |  |  | No qualitativ Study |  | Influence of osteopathy on the health-related quality of life of women after caesarean section. |
| Rodondi et al. (2019). |  | Not primarily osteopathic. |  |  |  | other topic |  | Primary care patients' use of conventional and complementary medicine for chronic low back pain. |
| Schleusener (2019). |  |  | Essay about statistics. |  |  | other topic |  | A vital warning... interested? |
| Seidner et Belz (2019). |  | Not fitting into the study profile. |  |  |  |  | other topic | Pediatric osteopathy: What registered pediatricians think about it - A qualitative interview study. |
| Ehmke (2018). |  |  |  | Different study design. |  | other topic |  | The identity of osteopathy in Germany - Search for traces of a professional self-conception. |
| Grill (2018). |  | Effectiveness of OMT. |  |  |  | No qualitativ Study |  | Influence of Osteopathic Treatment on Subacromial Impingement Syndrome (SAIS). |
| Hocher (2018). |  | Not fitting into the study profile. |  |  |  |  | other topic | The osteopathic treatment from the patient's point of view - Interviews with patients about their subjective well-being during osteopathic treatments. |
| Klapman et al. (2018). |  | Not an osteopathic study. |  |  |  | other topic |  | A snapshot of health information exchange across five nations: an investigation of frontline clinician experiences in emergency care. |
| Krause (2018). |  |  | Not fitting into the study profile. |  |  |  | other topic | Finding health. |
| Lohr et al. (2018). |  | Not an osteopathic study. |  |  |  | No qualitativ Study |  | Reliability of tensiomyography and myotonometry in detecting mechanical and contractile characteristics of the lumbar erector spinae in healthy volunteers.A42. |
| Mildenberger (2018). |  | Not fitting into the study profile. |  |  |  | other topic |  | Osteopathy in Germany before 1945. |
| Nier (2018). |  |  | Different study design. |  |  | other topic |  | Nutritional Knowledge: An integrative part of osteopathy in Germany? |
| Oringer (2018). |  | Effectiveness of OMT. |  |  |  | No qualitativ Study |  | Possible success of an individualized osteopathic treatment regarding the quality of life and the state of anxiety of a patient with a chronic lung sarcoidosis. |
| Rodondi et al. (2018). |  | Not primarily osteopathic. |  |  | No qualitativ Study |  |  | Primary care physicians' attitude and reported prescribing behavior for chronic low back pain: An exploratory cross-sectional study. |
| Samouh (2018). |  | Effectiveness of OMT. |  |  |  | other topic |  | Can the pregnancy rate after assisted reproductive technology be increased through osteopathic treatments? |
| Schötta (2018). |  |  | Not fitting into the study profile. |  |  | other topic |  | The mindful A.T. Still. |
| Steel et al. (2018). |  | Not fitting into the study profile. |  |  |  | other topic |  | The perceptions and experiences of osteopathic treatment among cancer patients in palliative care: a qualitative study. |
| Vaucher (2018). |  |  | Different study design. |  |  |  | other topic | The role of osteopathy in the Swiss primary health care system: a practice review. |
| Alcade-Rabanal et al. (2017). |  | Not an osteopathic study. |  |  |  | other topic |  | The gap in human resources to deliver the guaranteed package of prevention and health promotion services at urban and rural primary care facilities in Mexico. |
| Andrejcic (2017). |  |  | Different study design. |  |  |  | other topic | Mindfulness and its relevance to osteopathic practice. |
| Cummings (2017). |  |  | Osteopathy in the USA. |  |  | other topic |  | Meeting ACGME Standards Under a Unified Accreditation System: Challenges for Osteopathic Graduate Medical Education Programs. |
| Ducrestet al. (2017). |  | Not primarily osteopathic. |  |  |  | other topic |  | Complementary medicine use among general internal medicine inpatients in a Swiss university hospital. |
| Fischer (2017). |  | Book-Review. |  |  | other topic |  |  | The fascial distortion model: A medical concept - Praxiswissen Kompakt. |
| Herz (2017). |  |  | Different study design. |  |  | other topic |  | Resilience – How much stress can Austrian Osteopaths handle? |
| Huss et al. (2017). |  | Effectiveness of OMT. |  |  | No qualitativ Study |  |  | The influence of an osteopathic treatment at due date on the onset of labor within a 48-hour period. A randomized controlled trial. |
| Möckel (2017). |  | Interview. |  |  |  | No qualitativ Study |  | In dialog with Sandra Bartu. |
| Newby (2017). |  | Not an osteopathic study. |  |  |  | other topic |  | Practice characteristics that matter in the provision of health education services by primary care physicians. |
| Newiger (2017). |  | Not fitting into the study profile. |  |  |  | other topic |  | Osteopathy as current optional benefit of statutory health insurance companies. |
| Tschinkel (2017). |  |  | Different study design. |  |  |  | other topic | Osteopathy and Psyche. |
| Wentzel (2017). |  | Congress report. |  |  |  | No qualitativ Study |  | Review: 13th International Congress of the Osteopathy School Germany. |
| Aveni et al. (2016). |  | Not primarily osteopathic. |  |  |  | other topic |  | The Attitudes of Physicians, Nurses, Physical Therapists, and Midwives Toward Complementary Medicine for Chronic Pain: A Survey at an Academic Hospital. |
| Cummings (2016). |  |  | Not fitting into the study profile. |  |  |  | other topic | Osteopathic Students' Graduate Medical Education Aspirations Versus Realities: The Relationship of Osteopathic Medicine and Primary Care. |
| Gazea et al. (2016). |  | Lab-Study. |  |  |  |  | other topic | Definition of a critical spatiotemporal window within which primary cilia control midbrain dopaminergic neurogenesis. |
| Griesinger (2016). |  |  | Congress report. |  |  |  | other topic | Osteopathy focuses on children. |
| Hartmann et al. (2016). |  | Not primarily osteopathic. |  |  |  |  | other topic | Use of complementary and alternative medicine (CAM) by parents in their children and adolescents with epilepsy - Prevelance, predictors and parents' assessment. |
| Jutte (2016). |  | Not primarily osteopathic. |  |  |  |  | other topic | Naturopathy and Complementary Medicine in Medical Guidelines - Can European Standards Replace Guidelines for Naturopathy and Complementary Medicine? |
| Kayser et Harke (2016). |  | Effectiveness of OMT. |  |  |  | other topic |  | Manual medicine and osteopathic methods on the growing spine. |
| Licciardone et al. (2016). |  | Effectiveness of OMT. |  |  |  | other topic |  | Targeting patient subgroups with chronic low back pain for osteopathic manipulative treatment: responder analyses from a randomized controlled trial. |
| Mitschka et Wikus (2016). |  |  |  | Not fitting into the study profile. |  | other topic |  | Osteopathy in Austria - Legal basis and outlook. |
| Newiger (2016). |  |  |  | Not fitting into the study profile. |  | other topic |  | Occupational law for osteopathy. |
| No Author listed (2016). |  |  |  | No full-text available. | ? | ? | ? | Commitment of Austrian osteopaths to osteopathy. and to the Austrian Society for Osteopathy (OEGO). |
| No Author listed (2016). |  | Obituary. |  |  |  | No qualitativ Study |  | Obituary for Roger Seider. |
| Pauler (2016). |  | Congress report. |  |  | No qualitativ Study |  |  | The 20th European Symposium of Traditional Osteopathy. |
| Porges et Liem (2016). |  | Polyvagal theory. |  |  | other topic |  |  | The polyvagal theory in osteopathy. |
| Pöschl (2016). |  | Effectiveness of OMT. |  |  |  | No qualitativ Study |  | Interrater Reliability Study on the Temporal Bone. |
| Prediger (2016). |  | Osteopathic Initiative. |  |  |  | other topic |  | Osteopathic initiative for refugees. |
| Raith et al. (2016). |  | Effectiveness of OMT. |  |  | No qualitativ Study |  |  | General Movements in preterm infants undergoing craniosacral therapy: a randomised controlled pilot-trial. |
| Chartier-Kastler et al. (2015). |  | Not an osteopathic study. |  |  |  | other topic |  | Durable improvements in urinary incontinence and positive treatment response in patients with idiopathic overactive bladder syndrome following long-term onabotulinumtoxinA treatment: Final results of 3.5-year study. |
| Costello et al. (2015). |  | Not an osteopathic study. |  |  |  | other topic |  | Change in child abuse potential as a predictor of post-assessment child disruptive behaviors after participation in PACE. |
| Drew (2015). |  |  |  | Different study design. |  | other topic |  | Learning style, seat preference, and past profession: Predicting traditional osteopathic student achievement. |
| Fahlgren et al. (2015). |  |  | Patient-survey in Sweden. |  |  | other topic |  | Person-centered osteopathic practice: patients' personality (body, mind, and soul) and health (ill-being and well-being). |
| Füssel (2015). |  | Commentary. |  |  |  | No qualitativ Study |  | [Reply from Prof. Füessl]. |
| Hebert et al. (2015). |  | Effectiveness of OMT. |  |  |  | No qualitativ Study |  | Serious Adverse Events and Spinal Manipulative Therapy of the Low Back Region: A Systematic Review of Cases. |
| Hensel et al. (2015). |  | Effectiveness of OMT. |  |  | other topic |  |  | Pregnancy Research on Osteopathic Manipulation Optimizing Treatment Effects: the PROMOTE study. |
| Hohenschurz-Schmidt (2015). |  | Congress report. |  |  | No qualitativ Study |  |  | Fluids and Osteopathy - Congress of the Osteopathic School of Germany 2014. |
| Kladny (2015). |  | Not fitting into the study profile. |  |  | other topic |  |  | History of conservative spinal therapy. |
| Klein et al. (2015). |  | Not primarily osteopathic. |  |  |  | No qualitativ Study |  | Usage of Complementary Medicine in Switzerland: Results of the Swiss Health Survey 2012 and Development Since 2007. |
| Levin (2015). |  |  | Essay about a concept of a osteopathic university. |  |  | No qualitativ Study |  | Osteopathic University - a concept. |
| Mildenberger (2015). |  | Not fitting into the study profile. |  |  |  | other topic |  | Chiropractic in the Federal Republic of Germany from 1949 to 1975. |
| Müller (2015). |  | Congress report. |  |  | No qualitativ Study |  |  | The 19th European Symposium of Traditional Osteopathy. |
| Newiger (2015). |  |  |  | Article about a court decision. |  | No qualitativ Study |  | The decision of the Higher Regional Court Düsseldorf dated September 8, 2015. |
| Papperger (2015). |  | Effectiveness of OMT. |  |  |  | other topic |  | Osteopathic treatments on preborn children within their first 3 years: how content are parents with the effects of treatment? |
| Zehnle (2015). |  | Commentary. |  |  | other topic |  |  | [Fibromyalgia syndrome. Patients request osteopathy]. |
| Accorsi et al. (2014). |  | Effectiveness of OMT. |  |  |  | other topic |  | Effect of osteopathic manipulative therapy in the attentive performance of children with attention-deficit/hyperactivity disorder. |
| Gillemot (2014). |  | Not fitting into the study profile. |  |  |  | other topic |  | Treatment of infants with adjustment disorders - Stimuli for the education according to the GAIMH. |
| Ismail-Tsaous (2014). |  | Not fitting into the study profile. |  |  |  | other topic |  | Subsidization of osteopathic treatments: Insured and health insurance companies benefit. |
| Kaiser (2014). |  | Congress report. |  |  | No qualitativ Study |  |  | The 17th International Congress of the German Osteopathic Association. |
| Maurer (2014). |  | Report. |  |  |  | other topic |  | At the Summer Olympics as an Osteopath. |
| Mildenberger (2014). |  | History of chiropractic in Germany. |  |  | other topic |  |  | Chiropractic in Germany - the time before 1945. |
| Mueller et al. (2014). |  | Not fitting into the study profile. |  |  | other topic |  |  | Occupation-related long-term sensory training enhances roughness discrimination but not tactile acuity. |
| Newiger (2014) [1]. |  |  | Overview research institutions. |  |  | other topic |  | Osteopathic research institutions: an overview. |
| Newiger (2014) [2]. |  |  | Overview health insurance. |  |  | other topic |  | Osteopathy as an additional service of statutory health insurance companies. |
| Ostendorf (2014) [1]. |  |  |  | No full-text available. | ? | ? | ? | [Osteopath - an independent health profession?]. |
| Ostendorf (2014) [2]. |  |  |  | No full-text available. | other topic |  |  | [Osteopathy as an "independent healing profession". Should balneology specialists treat patients independently?]. |
| Psczolla (2014). |  | Commentary. |  |  | letter to editor |  |  | [Letter to GM Ostendorf: osteopath --an independent health professional?]. |
| Vogel (2014). |  |  |  | Not fitting into the study profile. |  | other topic |  | Osteopathy and naturopathy in Switzerland. |
| Carruzzo et al. (2013). |  | Not primarily osteopathic. |  |  |  | other topic |  | Offer and use of complementary and alternative medicine in hospitals of the French-speaking part of Switzerland. |
| v. Heymann et Buchmann (2013). |  | Not fitting into the study profile. |  |  |  | other topic |  | Structured curricular advanced training osteopathic methods. |
| Hormes et al. (2013). |  | Effectiveness of OMT. |  |  | Review |  |  | Physical therapeutic intervention of tinnitus. A systematic review. |
| Ismail-Tsaous (2013). |  |  | Different study design. |  |  | other topic |  | Osteopaths in Germany: Selected results of the 2013 osteopathy census. |
| Müller et Pietsch (2013). |  | Effectiveness of OMT. |  |  |  | No qualitativ Study |  | Comparison of gait training versus cranial osteopathy in patients with Parkinson’s disease: A pilot study. |
| No Author listed (2013). |  |  |  | Not fitting into the study profile. |  | other topic |  | Future and quality of osteopathy in Germany. |
| Prediger et Risch (2013). |  | Congress report. |  |  | No qualitativ Study |  |  | The 16th International Congress of the German Osteopathic Association. |
| Psczolla (2013). |  | Not fitting into the study profile. |  |  |  | other topic |  | New: structured curricular training “Osteopathic methods". |
| Pullen et al. (2013). |  | Training of medicial students. |  |  |  | other topic |  | Video-teleconferencing with medical students to improve exposure to child and adolescent psychiatry. |
| Goldenstein (2013). |  | Congress report. |  |  | No qualitativ Study |  |  | Brain and osteopathy – Congress of the Osteopathy School Germany in Berlin. |
| Salandi (2013). |  |  | Different study design. |  | No qualitativ Study |  |  | Psychosocial working conditions of German osteopaths. Cross-sectional study. |
