# Supplementary material for: Characteristics, opportunities, and challenges of osteopathy (COCO) in the perceptions of osteopaths in Germany, Austria, and Switzerland: a metasynthesis
Source: Eur J Med Res. 2024 Dec 18;29:586. doi: 10.1186/s40001-024-02199-3 (PMC11658149; doi:10.1186/s40001-024-02199-3)
Supplement: Supplementary file 2 — Additional file 2. [file 40001_2024_2199_MOESM2_ESM.docx]

Table S3: List of selected codes

| **List of codes** | **Memo** |
| --- | --- |
| Code system |  |
| Characteristics of osteopathy | Code is assigned if the IPs (Interviewpartners) make general statements about characteristics of osteopathy or make statements that do not fit into the subcategories. |
| Characteristics of osteopathy\Definition of osteopathy | Code is used for the personal definition of osteopathy of the IPs.  Anchor example: "as a mainly manual treatment method that brings the patient into (...) the best possible (...) not homeostasis, but homodynamic (...) in which he can then meet and adapt to all (...) challenges that his (...) life offers in the best possible way". (O3, item 40) [21] |
| Characteristics of osteopathy\Qualities of osteopathy | Code is assigned when IPs comment on specific qualities of osteopathy.  Anchor example: "I think it's important to take time for the patient. Many methods are becoming shorter and shorter in terms of consultation times and you no longer feel that you are being taken seriously. I think it's a strength of osteopathy that you really take this time and listen to the patient and give them the opportunity to explain themselves" (O1, item 100) [17] |
| Characteristics of osteopathy\Differentiation from other professions in the healthcare sector | Code is used when statements are made to differentiate osteopaths from other (medical) professional groups.  Anchor example: "And then this working with our hands differentiates us from acupuncture or Ayurveda or whatever. So I think it's a (...) it, you can't pin it down to one thing, it's the mixture that makes it and what makes osteopathy special." (O10, item 47) [21] |
| Characteristics of osteopathy\Patient profile | Code is used for rather general descriptions of the patients treated by the IPs.  Anchor example: "all people who come to me because I am in their catchment area (...) is every age group and every kind of problem (...) (um) patients who come a long way come to mainly because of (um) endocrinological, metabolic problems (...) gynecological problems (...) so that's where (...) my teaching and training focus has mainly settled in recent years." (O3, item 38) [21] |
| Characteristics of osteopathy\Limits of osteopathy | Code that is assigned when the limits of the osteopathy are mentioned.  Anchor example: "the limits of the method are (...) well, for me they are reached where, for example, surgery is needed or antibiotics or simply (...) so with more serious illnesses or with (...) so with irreversible structural disorders. (...) I absolutely see a limit (...) to osteopathy. (...) Not a limit in the sense that you can't continue treatment there, but where something else is simply needed. (...) And where we are at a different level today than in Still's time." (O10, pos. 77) [21] |
| Characteristics of osteopathy\Anchor figures | Code is assigned if the IPs make general comments about their anchor figures or make statements that indicate the influence of anchor figures.  Anchor example: "But there will always be people who really care about this innermost quality of osteopathy. (...) And just as osteopathy has developed from Still to Sutherland, Becker, Viola Frymann (...) and all their names (...), or Mitchell and Jim Jealous now (...), so it will continue to develop." (O5, item 84) [21] |
| Challenges of osteopathy | Code is assigned if the IPs make general statements about the challenges of osteopathy or make statements that do not fit into the subcategories. |
| Challenges of osteopathy\Identity problem | Code is used when IPs describe the inhomogeneity within osteopathy. This includes differences in the definition, teaching and practice of osteopathy.  Anchor example: "The identity issue, I would say, is the (is the) most important thing. Since we (...) as you will perhaps find out in the next or further interviews, where is the definition is the most difficult question and nobody can answer that for you. And if we can't answer that and don't work through it, how are we supposed to argue what we are if we don't know exactly ourselves." (O6, item 49) [21] |
| Challenges of osteopathy\Disagreements within the osteopathic community | Code is assigned when IPs report that there are problems/disagreements/discrepancies within their profession.  Anchor example: "Well, maybe a little bit, because this is always an issue with us on the board, yes, and (uh)(...) I don't know now, but I can just say that from me now, yes. (uh) That there is quite a gap between biodynamic osteopathy and (and um) structural osteopathy, yes. And (um) (...) and that's something that worries me a bit, because we had an expert meeting on the board some time ago and actually we want to, actually as well as possible, the philosophy of osteopathy, although I don't call biodynamic a philosophy, yes. But there are simply very clear facts, yes. And from (and, and) limits. (Um) So it's not something where you just kind of do magic. Not at all, yes. You can see exactly how the patient leaves, yes, from the treatment. (...) (Uh) And there are tendencies that I don't find pleasant, where you just want to have it all outside. I can still understand that from politicians and from doctors, because they just want evidence-based medicine. (...) But from osteopaths themselves, I find that very worrying and there is a tendency in Austria." (O7, item. 102) [21] |
| Challenges of osteopathy\Education | Code is assigned when IPs comment on issues in osteopathic training, but also on quality assurance.  Anchor example: Well, the way it is at the moment and the way osteopathy has developed, it is a paramedical profession. (...) And I don't think that we are better than the other paramedical professions, because osteopaths would have to be able to do more and do more. Osteopathy could already be (super) super and could actually (...) do a lot, but not the way it is currently practiced and not the way the training is currently and not the way the (...) self-image and self-image of osteopaths is currently. (O9, item 79) [21] |
| Challenges of osteopathy\Research | Code is used when IPs describe research work as a challenge for osteopathy.  Anchor example: "So perhaps also before the scientific validation of the treatment technique, although I think that's just another way (ne), now that I've done my study and you can see what peanuts come out of it (laughs) and what kind of mass it takes." (O8, item. 65) [21] |
| Challenges of osteopathy\Country-specific professional situation: Germany | Code is used when IPs report on the professional policy situation in Germany.  Anchor example: "I think one (an) essential point is the naturopath, that there is one. And that in Germany you actually have an almost better legal situation to work as an osteopath through the naturopath." (O10, item 97) [21] |
| Challenges of osteopathy\Country-specific professional situation: Austria | Code is used when IPs report on the professional policy situation in Austria.  Anchor example: "Many colleagues work as physiotherapists, they also charge for osteopathy as physiotherapy, and yes, they are refunded in this way. And as a result, (...) osteopathy is also a little (...) less in the focus than it should be. I've been working for 20 years now; I'm only writing osteopathic invoices. (...) But (...) of course (...) I understand the problem. If somebody has fewer patients (...) and (...) has to charge for (...) physiotherapy, I absolutely understand the situation. (...). But (...) these problems are of course (...) long-burning issues." (O5, item 78) [21] |
| Challenges of osteopathy\Country-specific professional situation: Switzerland | Code is used when IPs report on the professional policy situation in Switzerland.  Anchor example: ""So here in Switzerland, this, this implementation of the new Health Professions Act will certainly be a big challenge at first. Especially in German-speaking Switzerland with the integration of all these osteopaths who don't have a GDK title." (O4, item 95) [17] |
| Opportunities of osteopathy | Code is awarded when IPs make general comments about opportunities and possibilities for osteopathy.  Anchor example: "(thinking about it) I believe that osteopathy is um much more um further  is in demand. I believe that osteopathy has its place in the conservative umm (pondering) medicine umm will find, in all / I believe that to the example, in ten years' time there will no longer be any rehabilitation clinics without osteopaths working there." (Interview 04, item 174) [18] |
| COVID 19 | Code is used when IPs talk about the impact of the COVID-19 pandemic on their work.  Anchor example "I have to wear a mask." (O4, item 103) [21] |
